# B-cell and T-cell quantification in minor salivary glands in primary Sjögren’s syndrome: development and validation of a pixel-based digital procedure

**DOI:** 10.1186/s13075-016-0924-2

**Published:** 2016-01-20

**Authors:** Sebastian Costa, Sacha Schutz, Divi Cornec, Arnaud Uguen, Isabelle Quintin-Roué, Agnès Lesourd, Jean-Marie Berthelot, Eric Hachulla, Pierre-Yves Hatron, Vincent Goeb, Olivier Vittecoq, Jacques Olivier Pers, Pascale Marcorelles, Alain Saraux, Valérie Devauchelle-Pensec

**Affiliations:** Pathology Department, Brest University Hospital, Brest, France; Biology Department, Brest University Hospital, Brest, France; Rheumatology Department, Brest University Hospital, Brest, France; Pathology Department, Vannes General Hospital, Vannes, France; Rheumatology Department, Hôtel Dieu University Hospital, Nantes, France; Department of Internal Medicine, Nord-de-France University, Claude-Huriez Hospital, Lille, France; Rheumatology Department, Amiens University Hospital, Amiens, France; Rheumatology Department, Rouen University Hospital, Rouen, France; EA 2216-ESPRI 29, Brest University, Brest, France; Pathology Department and EA 4685, Brest University Hospital, Brest, France; Rheumatology Department and EA 2216-ESPRI 29, Brest University Hospital, Brest, France

**Keywords:** Digital quantification, B/T lymphocytes, Minor salivary glands, Sjögren’s syndrome

## Abstract

**Background:**

Evaluating lymphocytic infiltration of minor salivary gland biopsy in primary Sjögren’s syndrome is challenging. We developed and evaluated a digital method for quantifying B and T lymphocytes in whole minor salivary gland biopsy slides.

**Methods:**

Minor salivary gland biopsies were immunostained with anti-CD20/anti-CD3 antibodies using red/brown chromogens. Slides were digitised and spliced into mosaics of smaller JPEG format images in which red and brown pixels were counted. ImageJ Cell counter was used for validation. Agreement between the digital and manual methods was evaluated using Bland-Altman plots and the interclass correlation coefficient. External validation relied on the Chisholm-Mason, Tarpley, and focus-score methods.

**Results:**

Of 62 minor salivary gland biopsy slides, 61.3 % had a Chisholm-Mason grade ≥ III or a focus score ≥1. The number of pixels correlated well with manual cell counts (r = 0.95 for red pixels vs. B cell count and r = 0.91 for brown pixels vs. T cell count). Interclass correlation coefficients between digital and manual counts were excellent (0.92 for B/T cells). B-cell proportion showed a significant positive correlation with the focus score (Spearman’s coefficient 0.463, *p* < 0.0001). Median B-cell proportion was lower in minor salivary gland biopsies with Chisholm grades I–II (2.5 % (0.2–13.9)) than III–IV (30.0 % (15.5–45.2)) and increased with Tarpley’s class (1, 2.2 % (0.2–6.6); 2, 27.2 % (13.0–38.9); and 3–4, 48.5 % (29.4–56.4); *p* < 0.001 for all comparisons). Minor salivary gland biopsy B-cell proportion was also significantly correlated with several markers of clinical and biological activity of the disease, especially with markers of systemic B-cell hyperactivation.

**Conclusion:**

The digital procedure proved accurate compared to the reference standard, producing reliable results for whole tissue sections.

**Trial registration:**

ClinicalTrials.gov [NCT00740948]. Registered 22 August 2008.

## Background

Primary Sjögren’s syndrome (pSS) is a common chronic autoimmune disease characterised by lachrymal and salivary gland dysfunction due in part to lymphocytic infiltration and tissue destruction [1–5]. Ocular and oral dryness combined with severe fatigue are the main symptoms, and a significant proportion of patients have extraglandular manifestations. The current reference standard for diagnosing pSS is a specific pattern of focal lymphocytic sialadenitis in labial minor salivary gland biopsies (MSGBs), defined as the presence of one or more dense aggregates of ≥50 lymphocytes adjacent to apparently normal tissue [1, 6–9]. A focus score (FS) ≥1/4 mm^2^ was established for classification and diagnosis criteria [10].

Several other histological scores or grading systems are also used to describe and evaluate glandular involvement in pSS [11].

The cell subtypes and immune mediators relevant to the pathophysiology of pSS have been identified [12–15]. The presence and number of the relevant cell subtypes must be determined in MSGB infiltrates [16]. The mononuclear infiltrates contain T cells, B cells, macrophages, interdigitating and follicular dendritic cells, and natural killer cells. T and B cells predominate by far among the inflammatory cells, and the proportion of B cells increases with lesion severity and histopathological scores [17]. The most advanced lesions contain tertiary ectopic lymphoid structures, which may have germinal centres [18–20].

Thus, detailed characterisation of the MSGB infiltrates may be useful to classify patients into clinically relevant subgroups. The presence of germinal centres has already been proven to predict not only greater pSS severity, but also the development of malignant lymphoma [20, 21]. Among infiltrate features, the number and/or proportion of B cells may be of particular interest, for at least three reasons. The proportion of B cells is highest in advanced lesions and increases with histopathological scores, i.e. mirrors disease severity. The B-cell burden within the glands might influence the efficacy of anti-B-cell drugs such as rituximab. Finally, as with peripheral B-cell monitoring, B-cell monitoring in serial MSGBs might improve patient management during follow-up.

No validated method for B- and T-cell quantification suitable for use on an everyday basis is available. Many studies relied on the Cell counter plugin of ImageJ software (http://rsb.info.nih.gov/ij/plugins/cell-counter.html) to count B and T cells [22–24]. This technique, although very time consuming, can be considered the reference standard for counting cells within a region of interest [25]. However, no guidelines exist about the best method for assessing cell-type proportions within lymphocytic infiltrates. Recently introduced digital procedures allow whole-slide scanning followed by software-based image assessment. Standardisation is a major advantage of these procedures. The availability of a standardised method for evaluating lymphocytic infiltrate subtypes in patients with pSS would be valuable, most notably for monitoring treatment responses.

Our objective here was to design a digital procedure for quantifying B and T cells within MSGB infiltrates in patients with pSS. We assessed our procedure relative to established histological methods.

## Methods

### Patients

The randomised multicentre TEARS trial (Tolerance and Efficacy of Rituximab in primary Sjögren’s syndrome) was designed to evaluate the efficacy of rituximab in pSS [26]. It was approved by the appropriate ethics committee (Comité de Protection des Personnes Ouest VI), and all patients gave written informed consent before study enrolment. The protocol was registered on ClinicalTrials.gov [NCT00740948]. All patients fulfilled American European Study Group classification criteria. MSGB was performed routinely during the study at four of the 14 participating centres (Brest, Lille, Nantes, and Rouen), and the 62 formalin-fixed paraffin-embedded MSGBs thus obtained were sent to the coordinating centre in Brest.

### Conventional MSGB evaluation

For each MSGB, a single pathologist specifically trained in pSS recorded the histological findings into a centralised database. Chisholm-Mason grades [1] were dichotomised as 0, I, and II (0+) vs. III and IV (1+). The original version of Tarpley’s classification [27] was a 0 to IV (4+) scale with class 0 indicating normal salivary tissue and class IV (4+) a diffuse round-cell infiltrate with fibrosis completely destroying the lobular architecture. We used a previously published simplification into three groups: SS-I, mild lesions, 1+; SS-II, intermediate lesions, 2+; and SS-III, severe lesions, 3+ and 4+ [17]. The FS [8, 9] and germinal centre-like structures were assessed as previously described [8, 11, 19, 27].

Double-staining immunohistochemistry with anti-CD3 and anti-CD20 antibodies was performed on 4-μm thick tissue sections using the Benchmark XT® automated slide preparation system (Roche Diagnostics, Meylan, France), according to the manufacturer’s instructions. After deparaffinisation and pre-treatment with cell conditioner 1 (pH 8) for 8 minutes, immunostaining was performed using anti-CD3 antibody (polyclonal rabbit antibody, Dako, dilution 1/100, 32 minutes incubation at 37 °C) followed by revelation with a brown chromogen using the ultraView Universal DAB Detection Kit® (Ventana Medical Systems, Roche Diagnostics). The slides were heated at 95 °C for 8 minutes and the second immunostaining was performed using anti-CD20 antibody (monoclonal mouse antibody, clone L26, Dako, dilution 1/100, 20 minutes incubation at 37 °C) with red-chromogen revelation using ultraView Alkaline Phosphatase Red Detection Kit® (Ventana Medical Systems). After washing and counterstaining with one drop of haematoxylin for 12 minutes and one drop of blueing reagent for 4 minutes, the slides were removed from the immunostainer, washed in water with dishwashing detergent, and mounted.

### Slide digitisation and conversion

**Fig. 1:**
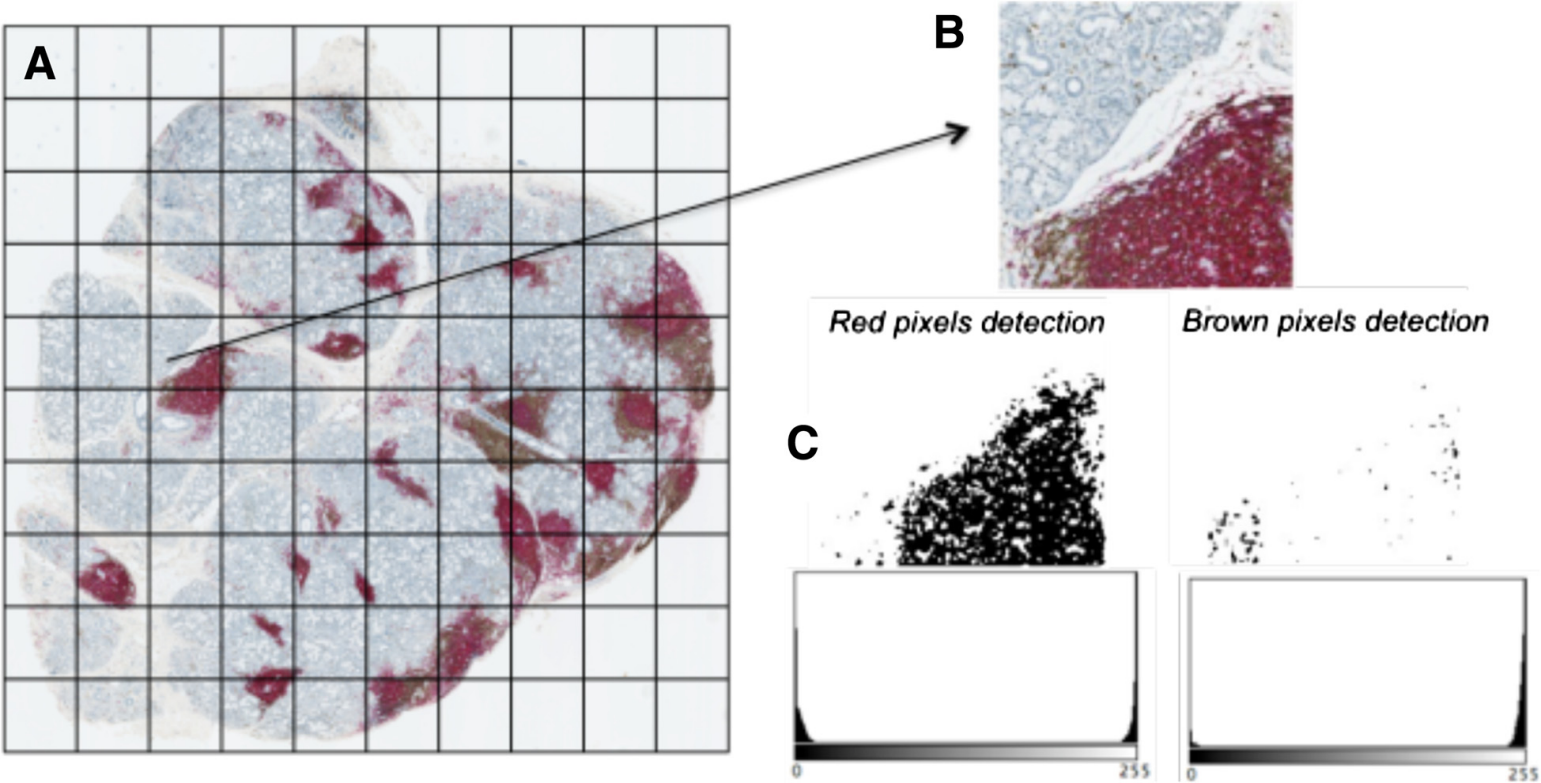
The digital pixel counting procedure. **a** A minor salivary gland biopsy section on a standard glass slide was scanned using the NanoZoomer HT Scan system. **b** The NDPI file thus obtained was divided into several JPEG format files, which restored the original image if reassembled. The JPEG format files were managed by the algorythm (source code, available on https://github.com/dridk/bgsa-ndpi). **c** The signal in a given pixel was partitioned using a linear unmixing algorithm with simple red/green/blue imagery

For each MSGB, the stained tissue slide was digitised using the NanoZoomer HT Scan system (Hamamatsu Photonics, Hamamatsu, Japan; http://www.hamamatsu.com/jp/en/index.html). Whole standard glass slides were scanned at 20-fold magnification (0.46 μm/pixel). The resulting virtual slides had a mean file size of 250 Mb (NDPI format). Each NDPI slide image was spliced to create a systematic mosaic of smaller JPEG images (about 1200 × 800 pixels per image with 9–318 images per gland and a mean of 42 images per gland), using Hamamatsu NDP.view2 viewing software (Fig. 1). The digital images covered the entire MSGB section. This way of proceeding ensured that no overlap occurred between the image fragments used to produce the mosaic; this point constitutes an advantage over manual acquisition.

### Digital procedure

Two of the authors (SS and SC) developed a digital procedure to quantify pixels corresponding to B and T cells in MSGBs. The source code, available on https://github.com/dridk/bgsa-ndpi, was written using Python 2.7 and licensed under GPL3. The same procedure was used to analyse the spliced NDPI image of each MSGB slide (Fig. 1). We used colour transformation to separate brown areas (indicating T cell pixels) and red areas (indicating B cell pixels). We used the YCbCr colour space, an alternative method for encoding red/green/blue (RGB) information that is well suited to JPEG format images. YCbCr contains one channel for luminance (Y) and two other channels for chrominance (blue and red). The red channel was selected; then, brightness and contrast were adjusted to obtain a binary (black and white) pattern in which red pixels were converted to black and all other colour channels to white. For the brown areas, we shifted hue and saturation into a different colour space (100, 200, 50) to convert the brown colour to red, which is more easily detected. We then applied the same process used for red. A histogram function counted the black pixels and white pixels. Thus, for each JPEG format image, we obtained the pixel counts for the red- and brown-stained areas.

### Validation of the digital procedure using manual B-cell and T-cell counts as the reference standard

**Fig. 2:**
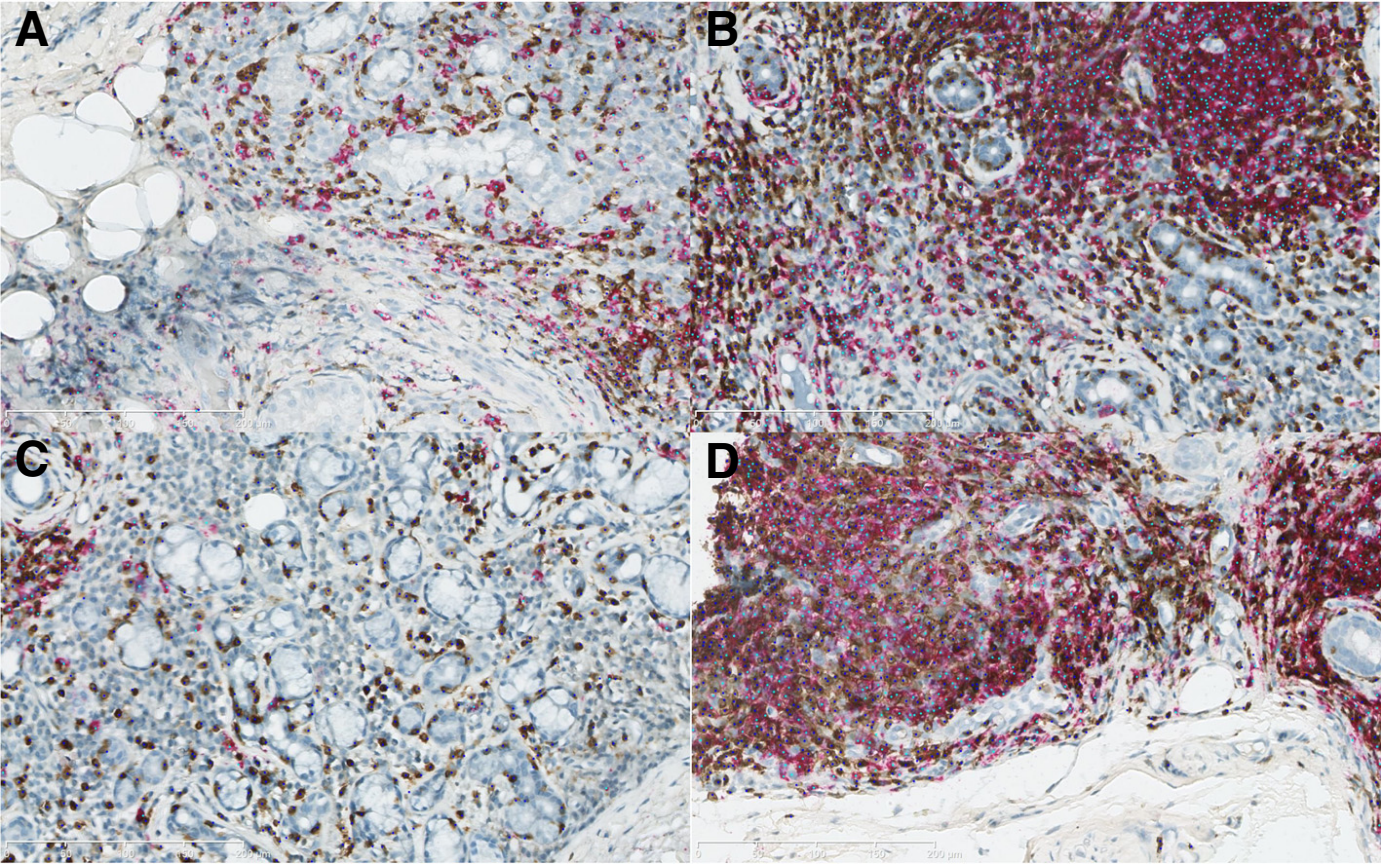
Micrographs of four JPEG format images showing the manual-count mask used by the ImageJ Cell counter. Stained B cells (in *red*) are marked with a dark blue dot and stained T cells (in *brown*) with a light blue dot. **a** First JPEG format image. Manual B-/T-cell counts: 135/361 (investigator 1) and 159/406 (investigator 2). Digital B-/T-cell count: 120/423. **b** Second JPEG format image. Manual B-/T-cell counts: 689/1067 (investigator 1) and 751/1089 (investigator 2). Digital B-/T-cell count: 532/1014. **c** Third JPEG-format image. Manual B-/T-cell count: 41/356 (investigator 1) and 50/426 (investigator 2). Digital B-/T-cell count: 36/410. **d** Fourth JPEG format image. Manual B-/T-cell counts: 469/860 (investigator 1) and 448/841 (investigator 2). Digital B-/T-cell count: 599/820

We selected a panel of 31 JPEG format images with various cell infiltrate densities (Fig. 2) and used the ImageJ Cell counter plugin to count T and B cells in each. At each click on a point of the image, a number whose colour indicated the cell type was displayed; there was one colour for T cells and another for B cells. The successive numbers thus obtained were tallied automatically. For each of the 31 images, this process was performed independently by two pathologists (AU and SC). The software ensured that each cell was counted only once and that all cells within a specified area were counted. Cells could be marked and re-examined to check that they were lymphocytes and not another cell type or a background artefact, without altering the cell count obtained so far.

Interclass correlation coefficients (ICCs) were computed to compare the B-cell and T-cell counts obtained by each pathologist. For each image and each cell type, the mean of the two manual counts served as the reference standard for evaluating our digital procedure. We recorded the time needed for the manual counts of each JPEG format image.

For each JPEG format image, we assessed correlations between manual B-cell count and red-pixel count and between manual T-cell count and brown-pixel count. We then converted the brown- and red-pixel counts (crude data) into digital counts of B and T cells. We summed the brown-pixel counts and the manual T-cell counts for the 31 JPEG format images and divided the first number by the second to obtain a coefficient of multiplication. The same procedure was then used to convert the red-pixel number into a digital B-cell count.

### Variation of B-cell and T-cell proportions according to histological examination

For each of the 62 MSGBs, we applied the digital procedure to each JPEG format image and then summed the digital T- and B-cell counts from all images. We then compared the whole-gland B- and T-cell digital counts thus obtained to the Chisholm-Mason grade, Tarpley class, and germinal-centre status.

### Correlation linking proportion of B cells in MSGBs to clinical and laboratory features of disease activity

To assess whether B-cell proportion as measured in MSGBs using our digital procedure might prove useful for monitoring patients with pSS, we assessed correlations linking B-cell proportion to the following markers for disease activity: oral dryness intensity as measured on a visual analogue scale (VAS); Eular Sjögren’s Syndrome Disease Activity Index (ESSDAI) [28]; unstimulated whole salivary flow; and biological markers for systemic B-cell activity (serum levels of IgG, anti-SSA antibodies, and kappa + lambda free-light chains measured using a commercial test (The Binding Site), according to the manufacturer’s instructions).

### Statistical analysis

We computed the ICCs to assess agreement between manual and digital B- and T-cell counts. We also generated Bland and Altman plots with their limits of agreement, defined as the mean of the differences ±1.96 standard deviations above and below the mean difference (known as the bias) [29]. With this method, about 95 % of normally distributed differences fall between the limits of agreement. The two measurement methods are considered equivalent if the difference between the highest and lowest limits of agreement is not clinically meaningful. To evaluate associations linking B- and T-cell proportions to histopathological parameters, we chose the Mann–Whitney test, Kruskal-Wallis one-way analysis of variance, or Spearman’s correlation coefficient.

Statistical analyses were performed using R (www.r-project.org) and GraphPad Prism (San Diego, CA, USA) software. Values of *p* < 0.05 were considered significant.

## Results

Details on the study population have previously been published (in Table 1, group II in [30]). Of the 62 MSGBs, 38 (61.3 %) had a Chisholm-Mason grade > III or a FS ≥1, 12 (19.3 %) contained germinal centres, 44 (71 %) had focal lymphocytic sialadenitis, 29 (46.8 %) had signs of non-specific chronic sialadenitis, and 4 (6.4 %) appeared normal. In the simplified Tarpley classification, 25 (40.3 %) were SS-I, 22 (35.5 %) SS-II, and 15 (24.2 %) SS-III.

**Table 1:** Validation and calibration of the digital procedure; 31 JPEG format files were chosen to represent several inflammatory infiltrate densities

JPEG format image	B cells (AU)	B cells (SC)	Red pixels	T cells (AU)	T cells (SC)	Brown pixels
ST5/1/gland_26_5.jpg	10	10	2656	98	88	6863
ST5/1/gland_26_8.jpg	25	24	1778	112	114	5755
ST5/1/gland_26_10.jpg	161	154	30,292	209	183	9368
ST5/1/gland_26_13.jpg	50	53	10,363	201	184	8956
ST5/1/gland_26_18.jpg	116	102	19,832	325	293	22,583
ST5/1/gland_26_21.jpg	166	162	27,671	278	240	12,576
ST5/1/gland_49_18.jpg	151	137	27,109	512	485	25,954
ST5/1/gland_71_30.jpg	109	129	24,932	384	383	27,830
ST5/1/gland_71_47.jpg	135	159	34,795	361	406	42,367
ST5/1/gland_71_48.jpg	132	114	24,067	303	269	19,527
ST5/1/gland_71_54.jpg	110	115	38,737	165	145	23,610
ST5/1/gland_71_62.jpg	241	216	95,764	150	126	15,149
ST5/1/gland_71_65.jpg	288	284	108,027	260	242	33,111
ST5/1/gland_71_83.jpg	181	167	51,012	211	179	30,594
ST5/1/gland_86_0.jpg	235	211	106,218	401	408	52,289
ST5/1/gland_86_2.jpg	121	118	40,319	522	516	88,540
ST5/1/gland_86_3.jpg	469	448	171,921	860	841	81,997
ST5/1/gland_87_16.jpg	689	751	153,788	1067	1089	101,444
ST5/1/gland_87_24.jpg	41	50	10,413	356	426	41,090
ST5/1/gland_87_28.jpg	157	147	54,422	613	547	63,898
ST5/1/gland_87_29.jpg	57	77	19,191	274	297	29,548
ST5/1/gland_89_0.jpg	117	135	37,489	130	103	17,342
ST5/1/gland_89_1.jpg	0	0	89	29	29	3567
ST5/1/gland_89_2.jpg	153	154	39,934	198	170	22,261
ST5/1/gland_89_3.jpg	2	2	324	10	9	1115
ST5/1/gland_89_4.jpg	0	0	6	12	15	1075
ST5/1/gland_89_5.jpg	24	21	3310	135	113	15,717
ST5/1/gland_89_6.jpg	1	1	131	19	17	2194
ST5/1/gland_89_7.jpg	1	1	88	30	31	4321
ST5/1/gland_89_8.jpg	1	2	113	64	69	6119
ST5/1/gland_89_9.jpg	11	9	909	273	297	26,749
Mean	128	128	36,635	276	268	27,210
Median	116	115	24,932	211	184	22,261
Q1–Q3	18–159	16–156	2217–40,126.5	121–358.5	108–394.5	7910–31,852.5

The ImageJ Cell counter plugin was used to count B and T cells in each file. For each of the 31 images, the process was performed independently by two pathologists (AU and SC). The third and the last columns indicate the number of pixels calculated with the digital procedure for B and T cells. *Q*

### Validation and calibration of the digital procedure

#### Agreement between the two pathologists

The mean manual counts obtained independently by the two pathologists (AU and SC) were 128 and 128, respectively, for B cells, and 276 and 268, respectively, for T cells (Table 1). The ICC was 0.99 (95 % confidence interval (CI) 0.987–0.997) for both cell types. Discrepancies were noted for dense infiltrates containing over 200 lymphocytes per JPEG format image. The reference standard for assessing the accuracy of our digital procedure was the mean of the counts obtained by the two pathologists. Manual counting took approximately 15 minutes (range 6–30 minutes) per JPEG format image.

#### Agreement and calibration between the manual and digital counts

**Fig. 3:**
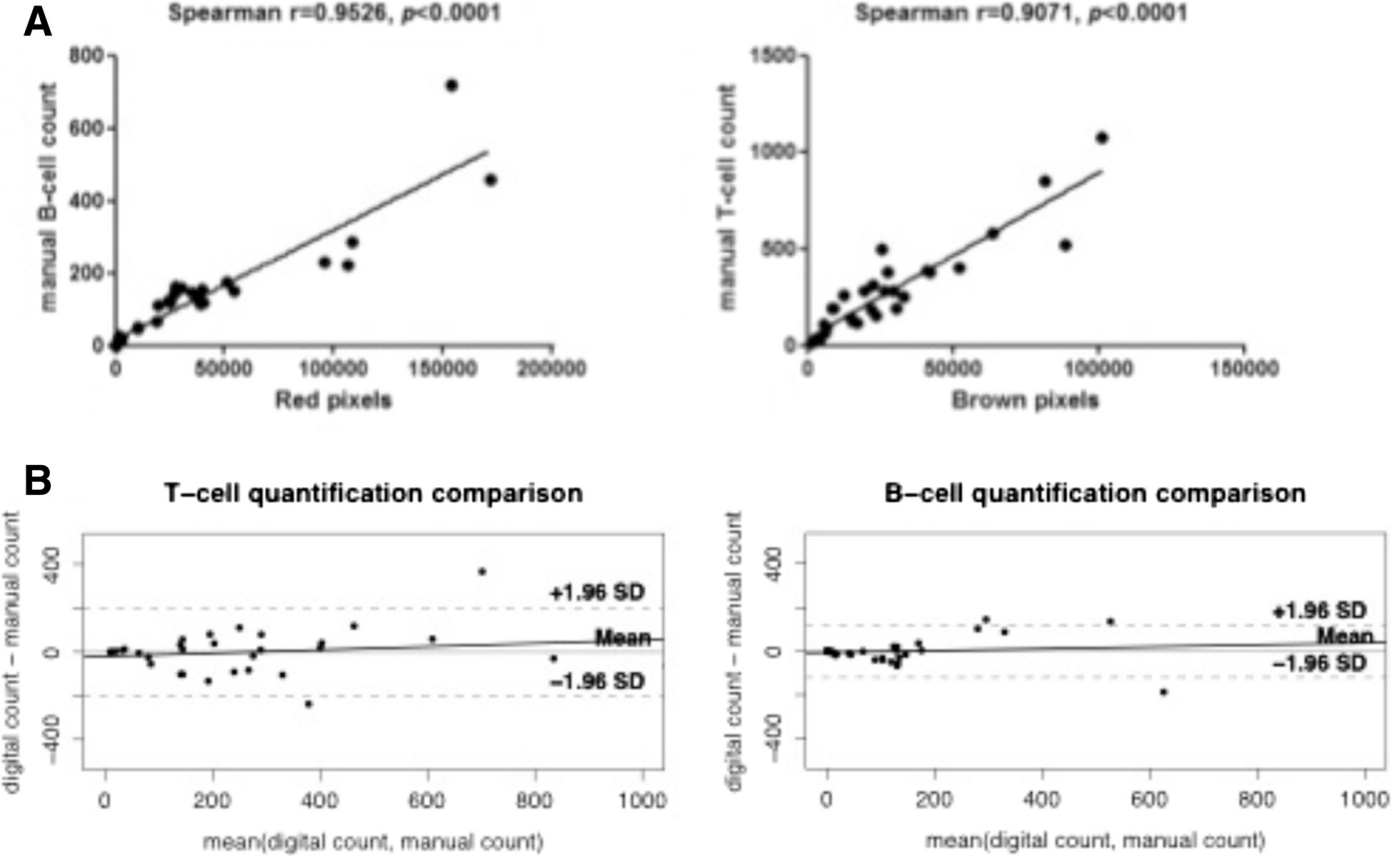
Comparison of manual and digital counts. **a** Relations between the numbers of red and brown pixels and the manual counts of B and T cells, respectively. **b** Bland-Altman plots of digital versus manual counts for 31 JPEG format images. There is no tendency to under- or overestimate counts lower than 200 cells. With counts greater than 200 cells, our in-house software slightly overestimated the manual counts. *SD* Standard deviation

Mean manual counts for the 31 JPEG images were 128 for B cells and 272 for T cells (Table 1). Mean digital counts were 36,635 for red pixels (B cells) and 27,209 for brown pixels (T cells). Manual and digital counts were closely correlated (Fig. 3). The factors that converted the red- and brown-pixel counts to B- and T-cell counts were 1/287 (128/36,635) and 1/100 (272/27,209), respectively. The ICC for manual and digital counts was 0.92 (95 % CI 0.84–0.96) for both B cells and T cells (*p* = 2.19 × 10^−14^ and *p* = 1.12 × 10^−14^, respectively). The Bland-Altman plot (Fig. 3c) showed no tendency to underestimate or overestimate cell counts lower than 200. For counts greater than 200 cells, the software tended to slightly overestimate the reference-standard count. Figure 2 shows three examples of B-cell and T-cell counts. Digital counting of the 31 JPEG format images took 3 minutes. Analysis of the 2648 JPEG format images for all 62 MSGBs took 180 minutes, yielding a mean of less than 3 minutes per gland. For manual counts, 15 minutes were needed per JPEG format image, i.e. 648 hours for all 62 MSGBs. Some JPEG format images contained no B or T cells or no gland parenchyma, as the mosaics included peri-glandular adipose tissue and even empty areas. Nevertheless, manual counting was far more time consuming.

### Associations linking B cells, T cells, and infiltrate severity

**Fig. 4:**
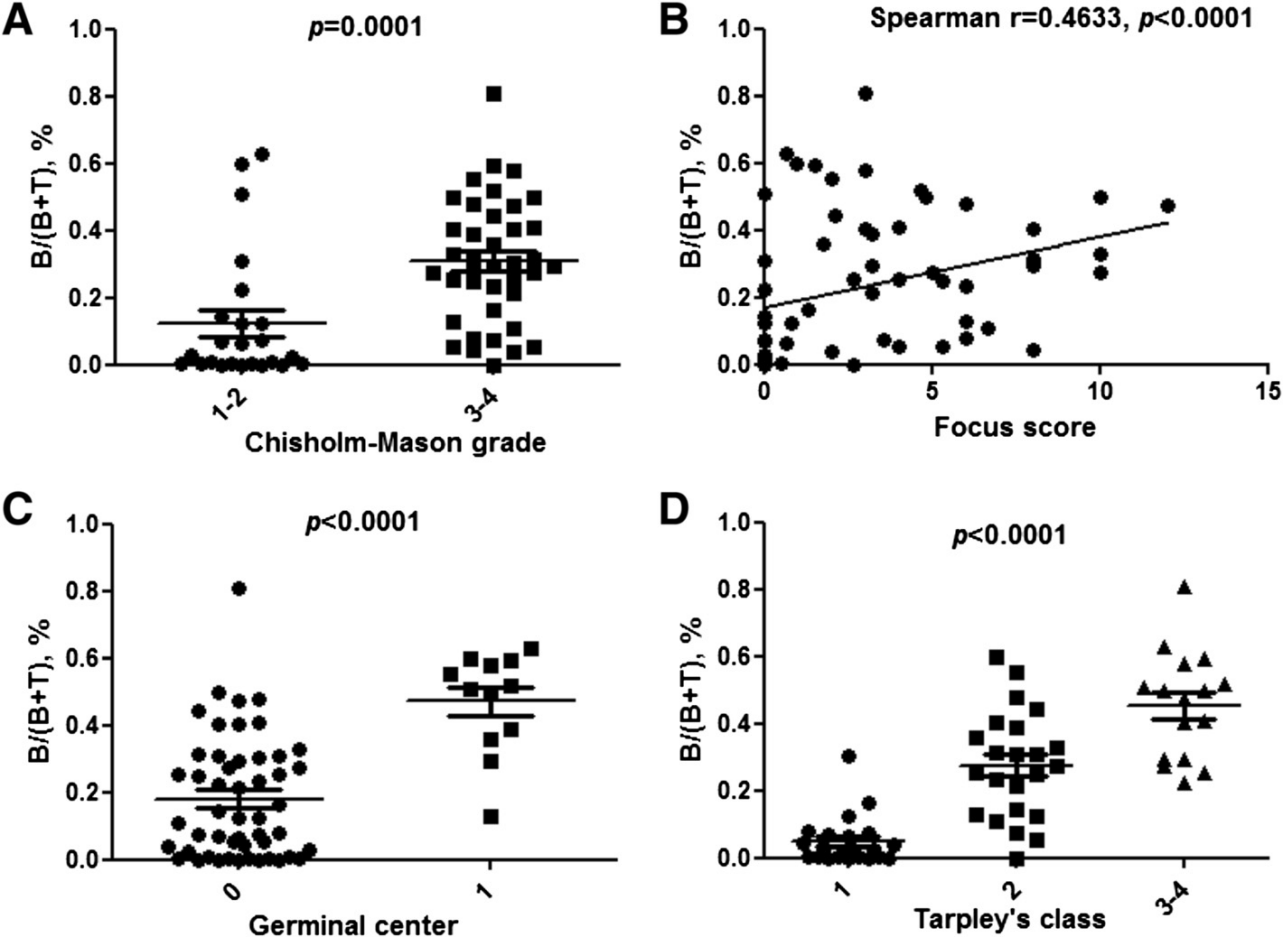
Association between proportion of B cells and infiltrate severity. The proportion of B cells (computed as B-cell count/(B-cell + T-cell count)) was related to four markers of infiltrate severity: Chisholm-Mason grade (**a**), focus score (**b**), presence of germinal centres (**c**), and Tarpley’s class (**d**). The proportion of B cells varied significantly with the values of these severity markers (*p* < 0.0001)

The mean area of tissue was 5.9 mm^2^ (1–50 mm^2^) and the median was 4 mm^2^. As B and T cells account for over 90 % of all cells in lymphocytic infiltrates, we defined the total lymphocytic infiltrate burden as the sum of the B- and T-cell counts [17]. We therefore computed the proportion of B cells as the B-cell count divided by the sum of the B- and T-cell counts. The proportion of B cells ranged from 0.01 % to 81 %; the median was 22.9 % (4.5–40.2). The proportion of T cells ranged from 18.9 % to 99.9 % with a median of 77.1 % (59.8–95.5). The relative proportions of B and T cells varied with the results of the histological parameters: B-cell proportions were higher in glands with worse histological parameters (Fig. 4). The proportion of B cells showed a significant positive correlation with the FS (Spearman coefficient 0.463, *p* < 0.0001). When we compared glands with Chisholm-Mason grades of I–II (n = 24) vs. III–IV (n = 38), we found that the median proportion of B cells was 2.5 % (0.2–13.9) vs. 30.0 % (15.5–45.2), respectively. The comparison of glands in Tarpley class I (n = 23), II (n = 23), and III–IV (n = 16) showed median proportions of B cells of 2.2 % (0.2–6.6), 27.2 % (13.0–38.9), and 48.5 % (29.4–56.4), respectively. Glands with (n = 12) and without (n = 50) germinal centres had median proportions of B cells of 51.4 % (36.6–58.9) vs. 12.3 % (1.9–30.6) (*p* < 0.001 for all comparisons).

### Associations linking proportion of B cells in salivary glands to clinical and laboratory markers of disease activity

**Fig. 5:**
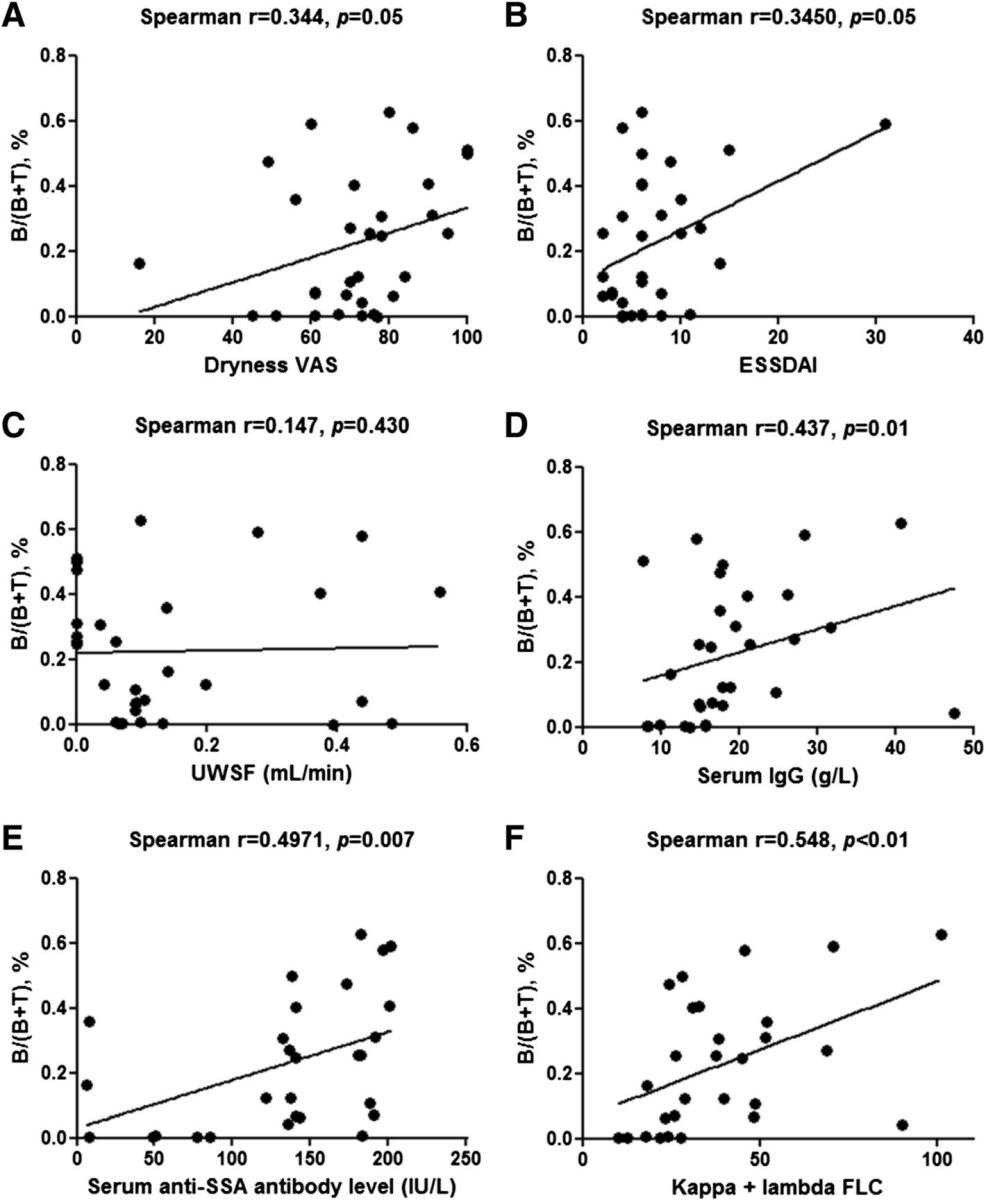
Correlations linking proportion of B cells to clinical and laboratory markers for disease activity. The proportion of B cells within minor salivary glands correlated with the intensity of oral dryness (visual analogue scale (*VAS*) score) (**a**), systemic activity (Eular Sjögren’s Syndrome Disease Activity Index; *ESSDAI*) (**b**), and biological markers for B-cell activity (serum IgG, anti-SSA antibody, and free light chains levels) (**d**, **e**, **f**), but not with unstimulated whole salivary flow (*UWSF*) (**c**)

The proportion of B cells in MSGBs correlated positively with the oral dryness VAS score (Fig. 5a), the ESSDAI (Fig. 5b), and laboratory markers of B-cell activity (serum IgG, anti-SSA antibody, and kappa + lambda free light chains levels (Fig. 5d, e, and f). Conversely, the proportion of B cells did not correlate with unstimulated whole salivary flow (Fig. 5c).

## Discussion

Our original digital cell pixel counting procedure proved fast and reliable for determining B- and T-cell proportion within salivary glands from patients with pSS. The digital pixel count correlated well with the manual cell count. The mean of red pixels was higher than the mean of brown pixels. This result suggests that the red chromogen has a larger area of staining or is more discriminable by the algorithm than the brown chromogen. This result was corrected by the conversion factor established on the panel of 31 JPEG format images chosen to represent several inflammatory infiltrate densities, including areas with no inflammatory cells and areas with high densities such as germinal centres.

The digital counts correlated significantly with well-established diagnostic and prognostic parameters such as the FS and Tarpley’s class. Furthermore, the significant correlations linking the digital counts to systemic disease activity (ESSDAI) and biological markers (e.g., anti-SSA antibody) provided external validation of our method. A major advantage of our digital procedure is that it assesses the whole tissue section instead of a limited number of microscopic fields or a selected region of interest. Manual cell counting was performed in most studies by applying the ImageJ Cell counter to serial images taken using image-acquisition systems connected to the microscope. Although these systems appear accurate, their use is time consuming and can result in overlap among images with some cells being counted more than once. Automated systems are associated with less intra- and inter-observer variability compared to manual counting methods. They make the counting procedure both less cumbersome and less subjective, thereby improving reproducibility and precision [31, 32]. Finally, developing an in-house procedure provides the opportunity to take into account and optimise all the factors that may influence the staining process.

However, the limitations of digital methods stem from the factors that affect assessments of the extent and intensity of immunohistochemical staining in general [30–34]. Staining can be influenced by transportation, fixation, and sectioning of the specimen; antigen retrieval; staining protocols; and control reagents. A challenge to the accuracy of digital automated systems is that the relation between the amount of antigen and the intensity of the signal is not always linear [31, 33]. As a consequence, the signal on the slide may not be strictly representative of the abundance of the antigen in the tissue section. Difficulties may arise in separating similar chromogens or different signals that overlap in space. The optical signal from each chromogen must be isolated to ensure accurate counting. When developing our method, we experienced substantial difficulty in separating brown from red using the RGB digital colour space, due to overlap among the spectral profiles. We therefore used a different digital colour format in the RGB model (Hue-Saturation-Value), which improved discrimination between red and brown. The RGB approach is less precise than multispectral analysis. Multispectral cameras acquire stacks of images at multiple wavelengths and allow the determination of precise optical spectra for each pixel [33, 34]. However, multispectral requires expensive equipment. Even though powerful image analysis systems are emerging, which also take into account morphology criteria, our results suggest that pixel area quantification is strongly correlated to manual count, histological and clinical factors.

Finally, we have shown that digital counting of stained cells throughout an entire tissue section was accurate and avoided the drawbacks of manual counting such as uncertainties about the number of cells to be counted (e.g., 100, 200, 500, or 1000?) and where to count the cells (e.g., germinal centres, sites of highest cell density, or sites of lowest cell density?).

## Conclusions

To our knowledge, we have designed the first pixel-based method that uses the RGB approach to separately count B and T cells in MSGBs from patients with pSS. In addition to saving time, our method has the major advantages of being standardised and automated. We actually use it on a prospective research basis and wish to validate it externally with other centres, given the variation in staining methods, as some hospital clinical laboratories may prepare tissues under different conditions.

The increased accuracy of digital B- and T-cell counts compared to manual counts may help to divide patients with pSS into clinically relevant subgroups, thereby optimising treatment decisions by improving the prediction of long-term outcomes and treatment responses.

## Abbreviations

CI: Confidence interval
ESSDAI: Eular Sjögren’s Syndrome Disease Activity Index
FS: Focus score
ICC: Interclass correlation coefficient
MSGB: Minor salivary gland biopsy
pSS: Primary Sjögren’s syndrome
RGB: Red/green/blue
VAS: visual analogue scale
